# Lupus Exedens, with the History of a Case Successfully Treated by the Use of the Solar Ray Cautery

**Published:** 1883-03-20

**Authors:** O. V. Thayer


### LUPUS EXEDENS, WITH THE HISTORY OF A CASE SUCCESSFULLY TREATED BY THE USE OF THE SOLAR RAY CAUTERY.

BY O. V. THAYER, M.D.

**Read before the San Francisco County Medical Society.**

Lupus is a chronic inflammation of the skin, appearing in the shape of external tubercles, of different sizes, singly or in clusters, of a livid red color and indolent character, followed either by ichorous ulcers, which become covered with brownish and usually very adherent scales (lupus exedens), or by extensive changes in the structure of the skin, but without ulcerations (lupus non-exedens).

This disease is generally confined to the face. It may attack at once or in succession several regions of the body. The two varieties which have been indicated are very distinct in their external appearances, and, also, require different modes of treatment. Lupus exedens is generally developed on the alæ, or tip of the nose. It makes its appearance as a small tubercle of a dusky red color, whose progress is usually tardy; sometimes as a chronic inflammation of the mucous membrane of the nasal fossæ. A thin scab or crust then forms at the opening of the nostrils. This is removed and a second and thicker one succeeds. An ulcer, in fact, has been formed, and soon extends to the alæe of the nose.

Under other circumstances, a livid purple tint and some swelling of the end of the nose are the first symptoms of the disease observable.

The redness increases, a superficial sore is formed, which becomes covered with a scab and the ulcer extends in depth. It may be confined to only one of the alæ, which swells, becomes painful and of a purple hue, while the other remains free from disease. A slight ulcer then forms and becomes covered with a little scab. This the patient commonly picks off, when it is replaced by a thicker one, under which the ulcerative process continues to go on, the scab being found to increase in thickness every time it is removed. The skin, and, occasionally, the cartilage, is silently destroyed and an ulcer of bad character, from which a fetid, seropurulent discharge is poured out, is at length discovered, as if by accident, established under the scab. The ravages committed by this disease vary extremely.

Almost the whole of the nose disappears in one instance, and the point only suffers a little in another, in which case it often looks as if a piece had been removed by a cutting instrument.

When such ulcers have been arrested and healed up, new tubercles occasionally form on or near the cicatrices, and the parts which had been spared originally may be entirely destroyed by a renewal of the ulcerative process, and even the whole nose and septum may vanish before its destructive influence.

Sometimes, if the disease is interfered with, it seems to acquire new energy.

Incrustations, which are attended with acute pains and grow very thick, in the course of a few days form in the interior of the nasal fossæ, whence a puriform fluid distils, and the point of the nose is rapidly destroyed.

The disease seems, every now and then, to be advancing towards recovery, when the part that was almost cicatrized turns to a livid red, is attacked anew with painful ulceration and is covered with a thick scab, under which the destructive inflammation makes rapid progress.

In lupus exedens of the skin of the nose, the mucous membrane of the nasal fossæ is almost always affected with chronic inflammation. In some rare cases the septum is even destroyed before the outer surface of the nose is implicated.

The tubercles of lupus exedens are occasionally evolved near the commissure of the lip, ending in partial destruction of the parts by the shrinking of the cicatrices. When the disease gets well the opening of the mouth is apt to be considerably diminished.

The lower eyelid is occasionally attacked, ending in great deformity, the lid receding from the eyeball. The eyeball, in this case, being imperfectly protected, inflames, the conjunctiva thickens, the cornea loses its transparency and, by-and-by, becomes so dim that blindness follows.

The cicatrices which follow the healing of lupus ulcerations often result in the forming of white bands, stretching from the parts where the mischief began to those in the vicinity, and similar in appearance to the cicatrices that follow extensive burns.

Lupus exedens often continues for years, committing frightful ravages without the general health appearing to suffer to any great extent.

*Lupus non-exedens* does not ulcerate; but, on the contrary, the skin undergoes great structural change independent of ulceration. But I do not deem it advisable to discuss this variety of the disease in this paper.

Causes.—Lupus is, happily, a disease of rare occurrence. It seems more common in the country than in large towns, and to attack women more frequently than men.

Low, damp localities, wanting in sunlight, with poor, badly cooked food, predispose to this disease. Scrofulous children are, of all individuals, the most obnoxious to its attacks, yet it undoubtedly occurs among the robust, who have lived in the enjoyment of good health. It rarely shows itself at the age of fifty. The disease is not contagious, and is seldom seen in the better class of society.

Prognosis. Under ordinary treatment, lupus is always a very obstinate disease. Months and even years elapse before it yields to treatment. The earlier the period of its existence the more hope of success as to treatment prescribed.

So long as the cicatrices remain soft, bluish, and convey to the finger something of a feeling of fluctuation, and so long as they are surrounded with tubercles of different sizes, there are grounds to apprehend renewed attacks of erosive inflammation, in which case the tubercles ulcerate and the cicatrices already formed are not long in again becoming open.

TREATMENT: The first indication in commencing the treatment of lupus is to endeavor to modify the general condition by appropriate remedies. The patient should at once be placed under the very best of hygienic influences and proper regimen. The system should be built up, as it were. The disease itself is, at the same time, to be combatted by such external and internal remedies as appear to exert a salutary influence in the development and progress of tubercles and the ulcerative process. Among these are the preparations of iron, iron with bitter compounds, preparations of iodine, mineral waters, sulphur baths. The bath of pure hot water is of more benefit in my estimation than medicated ones.

These should be used often and regularly, two or three times a day. The food should be of good quality, well cooked, and taken every six hours. A residence in a dry and bracing atmosphere is a powerful modifier of the constitution.

Among local applications to the ulcerating surfaces, caustics have generally been relied upon—such as the nitrate of silver, potassa fusa, butter of antimony, super-nitrate of mercury, arsenical powders and paste, the actual cautery, and last, but not least, the Solar Ray Cautery.

When the disease is extensive, the cauterization should be done with great caution. It should be confined to a single part and extended, successively, to the whole of the affected surfaces. When the ulcers are covered with scabs, they must be remove l or got rid of by means of poultices.

During the treatment patients should avoid exposure to heat or rigorous cold and dampness. For want of attention to this simple precaution, cicatrices that appeared sound have frequently been seen to open out afresh. When the disease is accompanied with any evident functional disturbance, this must be remedied by appropriate means.

Having great confidence in the curative qualities of the Solar Ray Cautery, I determined to treat the first decided case that came under my observation by this means. I had not long to wait an opportunity.

Mrs. B——, aged 40, of Nevada, consulted me in the month of June last. She was suffering from a disease of the nose and upper lip, which made its appearance two years before, commencing in the septum of the nose, resembling an ordinary cold-sore. In the following September the disease had extended into the anterior nares, alæ, tip of the nose and upper lip. The skin of the nose seemed spongy, not unlike a pin-cushion. A pin or needle could be thrust into it without pain; a little blood would follow the pricking, with a slight oozing of watery fluid afterward.

In December following the patient came to this city for medical treatment. At this time the disease covered the right side of the nose, extending upward one-half the distance to the and down the lip near its margin. She was under treatment from this time to the next April, receiving, however, but little benefit.

At times the disease seemed to be improving, but soon to be followed by an aggravation of all the symptoms. Hard lumps, as large as a pea or bean, formed under the skin, which soon softened and ulcerated, discharging pus, to be followed sooner or later by a thick scab. From December to April many remedies were applied to the diseased parts. Some of them produced the most intense pains, lasting for hours. She informed me that the greatest relief and benefit was received from the use of an ointment prescribed by an old woman, the composition of which she was ignorant of.

At the time she came under my care the disease was confined mostly to the upper lip, directly under the nose, which was occupied by ulcers secreting pus. The anterior nares were red, the mucous membrane extremely vascular and thickened. The skin in the immediate neighborhood of the disease had a dark bluish tint and roughened appearance. This was the status of the disease after two years of unsuccessful treatment by physicians of this city and elsewhere.

With a powerful lens with a focal diameter of three lines, having a clear sky, an unobstructed sunlight, (the great essentials in the success of the Solar Ray Cautery,) I most thoroughly cauterized the diseased surface. This was accomplished in three minutes time. The cauterization was not very painful while using the lens, although I burned the skin to a crisp. All pain ceased immediately after the removal of the glass. This has been my experience in nearly all of the cases operated upon with the Solar Ray.

I dressed the burned surface with Zinc Ointment, over which was applied a compress wet in a 5% solution of carbolic acid. The next day there was more or less swelling of the lip. The parts seemed tender and inflamed. Thirty-six hours after the operation an improved condition was visible. The discoloration of the nose and lip disappeared rapidly from day to day, and within less than two weeks the patient presented herself at my office with the ulcerative surfaces most thoroughly healed, and, as you may well anticipate, a very grateful and happy patient.—*Pac. Med. and Surg. Journal.*

